# Supplementary material for: Resolving the full spectrum of human genome variation using Linked-Reads
Source: Genome Res. 2019 Apr;29(4):635–45. doi: 10.1101/gr.234443.118 (PMC6442396; doi:10.1101/gr.234443.118)
Supplement: Supplemental Material [file supp_gr.234443.118_Supplemental_longranger-2.2.zip › longranger-2.2.tar.bz2!/lr2.2-public/lib/stan/cmdstan/stan/doc/api/html/agrad_2fwd_2matrix_2sum_8hpp_source.html]

Stan: src/stan/agrad/fwd/matrix/sum.hpp Source File


|  |  |
| --- | --- |
|  | Stan  2.5.0  probability, sampling & optimization |


- Main Page
- Related Pages
- Namespaces
- Classes
- Files

- File List
- File Members

All Classes Namespaces Files Functions Variables Typedefs Enumerations Enumerator Friends Macros Pages

- src
- stan
- agrad
- fwd
- matrix

sum.hpp

Go to the documentation of this file.

1 #ifndef STAN\_\_AGRAD\_\_FWD\_\_MATRIX\_\_SUM\_HPP

2 #define STAN\_\_AGRAD\_\_FWD\_\_MATRIX\_\_SUM\_HPP

3

4 #include <vector>

5 #include <stan/math/matrix/Eigen.hpp>

6 #include <stan/agrad/fwd/fvar.hpp>

7

8 namespace stan {

9  namespace agrad {

10

11  template <typename T, int R, int C>

12  inline

13  fvar<T>

14  sum(const Eigen::Matrix<fvar<T>,R,C>& m) {

15  fvar<T> sum = 0;

16  if (m.size() == 0)

17  return 0.0;

18  for(unsigned i = 0; i < m.rows(); i++) {

19  for(unsigned j = 0; j < m.cols(); j++)

20  sum += m(i,j);

21  }

22  return sum;

23  }

24  }

25 }

26 #endif

stan::agrad::fvar

**Definition:** fvar.hpp:13

stan::agrad::sum

fvar< T > sum(const Eigen::Matrix< fvar< T >, R, C > &m)

**Definition:** sum.hpp:14

fvar.hpp

Eigen.hpp


---

[ Stan Home Page ]

*© 2011–2014,
Stan Development Team.*
